# The role of data and safety monitoring boards in implementation trials: When are they justified?

**DOI:** 10.1017/cts.2020.19

**Published:** 2020-03-05

**Authors:** Kevin Fiscella, Mechelle Sanders, Tameir Holder, Jennifer K. Carroll, Amneris Luque, Andrea Cassells, Brent A. Johnson, Stephen K. Williams, Jonathan N. Tobin

**Affiliations:** 1 Department of Family Medicine, University of Rochester Medical Center, Rochester, NY, USA; 2 Clinical Directors Network, Inc. (CDN), New York, NY, USA; 3 Department of Family Medicine, University of Colorado, Boulder, CO, USA; 4 Department of Medicine, University of Texas Southwestern Medical Center, Dallas, TX, USA; 5 Department of Biostatistics and Computational Biology, University of Rochester Medical Center, Rochester, NY, USA; 6 Department of Medicine, New York University, New York, NY, USA; 7 Center for Clinical and Translational Science, The Rockefeller University, New York, NY, USA

**Keywords:** Clinical trials data monitoring committee, implementation science, data and safety monitoring, delivery science, translational medical research, National Institutes of Health, clinical trials as topic

## Abstract

The National Institutes of Health requires data and safety monitoring boards (DSMBs) for all phase III clinical trials. The National Heart, Lung and Blood Institute requires DSMBs for all clinical trials involving more than one site and those involving cooperative agreements and contracts. These policies have resulted in the establishment of DSMBs for many implementation trials, with little consideration regarding the appropriateness of DSMBs and/or key adaptations needed by DSMBs to monitor data quality and participant safety. In this perspective, we review the unique features of implementation trials and reflect on key questions regarding the justification for DSMBs and their potential role and monitoring targets within implementation trials.

As implementation scientists, we became interested in the purpose and role of data and safety monitoring boards (DSMBs) for implementation trials while conducting effectiveness-implementation trials [1,2]. Currently, we are conducting an implementation trial of evidence-based interventions to reduce cardiovascular risk factors [1]. This trial is part of “ImPlementation REsearCh to Develop interventions for People Living with HIV” (PRECluDE – RFA-HL-18-007): a National Heart, Lung, and Blood Institute (NHLBI)-funded study of implementation strategies for evidence-based interventions to reduce cardiovascular or pulmonary risk for people living with human immunodeficiency virus (HIV).

When we reviewed the DSMBs’ published literature, we found no articles that specifically discussed DSMBs for implementation trials. In this perspective, we reflect on this topic beginning with DSMBs’ purpose and history as well as the National Institutes of Health (NIH) and the NHLBI requirements, DSMBs’ relevance to implementation trials and whether DSMBs should be universally required. Additionally, we include a discussion of expanding the concept of individual participant risk to include risk to organizations and staff. We conclude with suggestions for implementation issues and outcomes that might be considered for advancing the field and enhancing the safety not only for patients, but also for organizations, settings, and staff participating in studies to implement evidence-based practices.

## DSMB Purpose, History, and Policies

DSMBs are committees of independent members with methodological and content expertise relevant to the particular trial that conduct interim monitoring, analysis, and oversight [3]. Thus, NIH policy requires DSMBs where there is heightened risk to *individual* participants. Notably, only individuals are addressed by The Common Rule, US 45 Code of Federal Regulations 46.102(e) [4]. The primary purpose of a DSMB is to ensure the safety of study participants [5] where study participants are generally limited to individuals who are directly impacted by the intervention. A secondary purpose is to protect and preserve data quality in order to safeguard the interests of participants and to produce reliable scientific findings that justify ongoing risk to participants [6]. The DSMB's role in conducting periodic benefit–risk assessments and their authority to recommend trial termination distinguishes them from other research oversight and advisory groups [7].

Although DSMBs may issue recommendations to improve participant protections and/or improve data quality and trial integrity, trial stoppage decisions are typically based on: (1) individual participant safety concerns; (2) overwhelming benefit; or (3) futility. DSMBs have a unique role in trial monitoring that is different from other oversight groups, for example, institutional review boards, ethics committees, or trial steering committees, in their access to unblinded interim results [7]. One of the earliest DSMBs was established in the late 1960s to monitor the University Group Diabetes Project following concerns about drug safety and adequate monitoring [8]. Subsequently, the Greenberg Report to NHLBI recommended requiring DSMBs for large clinical trials [9].

Current NIH policy provides latitude in appointment of DSMBs based on risk to participants:

All clinical trials require monitoring–Data and safety monitoring is required for all types of clinical trials…Monitoring should be commensurate with risks– The method and degree of monitoring needed is related to the degree of risk involved. A monitoring committee is usually required to determine safe and effective conduct and to recommend conclusion of the trial when significant benefits or risks have developed or the trial is unlikely to be concluded successfully…Monitoring should be commensurate with the size and complexity. Monitoring may be conducted in various ways or by various individuals or groups, depending on the size and scope of the research effort. These exist on a continuum from monitoring by the principal investigator or NIH program staff in a small phase I study to the establishment of an independent data and safety monitoring board for a large phase III clinical trial [10].

NIH defines a phase III clinical trial as a, “study to determine efficacy of the biomedical or behavioral intervention in large groups of people (from several hundred to several thousand) by comparing the intervention to other standard or experimental interventions as well as to monitor adverse effects, and to collect information that will allow the interventions to be used safely” [11]. Arguably, most implementation trials do not fit this definition because the usual intent of implementation trials is less on evaluation of individual effectiveness and safety, although there are potential exceptions for some hybrid trials that primarily evaluate effectiveness during implementation [12].

Current NHLBI policy is more expansive. NHLBI requires DSMBs “for all clinical trials that involve: investigation of a research question having direct implications for clinical care and/or public health (including all phase III trials), and/or a high-risk intervention, and/or a highly vulnerable population” [13]. Further, NHLBI requires DSMBs for multicenter trials and/or trials conducted under a contract or cooperative agreement [13]. Thus, these implementation trials funded by NHLBI must have DSMBs regardless of risk to individual participants. However, NHLBI policy is silent regarding the explicit purpose of this heightened monitoring in these contexts, much less what should be monitored during implementation trials.

## Are DSMBs Relevant to Implementation Trials?

The term “implementation” does not appear in NIH's “Important Clinical Trial-Related Terms.” Nonetheless, implementation research is defined by NIH “…as the scientific study of the use of strategies to adopt and integrate evidence-based health interventions into clinical and community settings to improve individual outcomes and benefit population health” [14]. Thus, an implementation trial uses a clinical trial design to evaluate strategies to adopt and integrate evidence-based interventions into practice. In contrast, pragmatic trials assess the effectiveness of interventions under real-world conditions [15]. Implementation trials often assess both effectiveness and implementation to varying degrees, that is, hybrid trials [12]. In implementation trials, the question is which strategies promote uptake of these evidence-based interventions, under what circumstances and why and whether findings of effectiveness can be embedded and sustained in real-world settings. Given that implementation trials often involve the use of evidence-based interventions, the risk to individual participants is often minimal, approximating the same level of risk associated with the delivery of routine clinical care [16]. Based on NIH guidance, DSMBs would not be generally required for clinical trials involving minimal risk.

The unique features of many implementation trials hinder the use of DSMB stopping rules. Primary implementation outcomes (e.g., acceptability, adoption, appropriateness, feasibility, implementation costs, reach, penetration, and sustainability) are commonly assessed using mixed methods [16]. The use of stepped wedge designs often limits longitudinal assessments to existing data. The “messiness” of multiple measures that are qualitatively and quantitatively assessed hinders the development of simple stopping rules. Effectiveness is often assessed in implementation trials using existing data (e.g., blood pressure reading or laboratory data) from electronic medical records rather than from direct assessment of individual participants. Adverse events for individual participants, resulting from the evidence-based intervention, are often not routinely collected. Among oversight groups, DSMBs have a unique role in their access to potentially unblinded results. However, this role is limited in the context of an implementation trial, where assessments often focus on group-level effectiveness and implementation processes, rather than individual-level, relative, benefit-to-harms. Many implementation trials do not involve the collection of sufficient, actionable, real-time data needed to inform DSMB recommendations for trial termination based on individual harm, let alone overwhelming benefit or futility.

## What Is the Role of DSMBs in Implementation Trials?

Based on the principle that the intensity of monitoring be commensurate with risk, it is reasonable to question whether DSMBs are generally relevant for monitoring participant safety in implementation trials. Potentially, this requirement runs the risk that participant protections and monitoring result in “over protection of the rights and interests of patients in some cases and under-protection in others?” [17] Thus, implementation trial participants are potentially overprotected, while patients exposed to major changes in health system policies or use of off-label medications might be under-protected.

When it comes to implementation trials, NHLBI policy does not clearly articulate what DSMBs should be monitoring and under what circumstances, how such risks should be monitored (rather than by whom) and what specific risks to individual participants or the trial itself warrant more intensive monitoring. Should DSMBs for implementation trials shift their primary focus from monitoring benefit–risk for participants to monitoring data quality and trial integrity? If so, how does this requirement translate into stopping rules for an implementation trial? Should implementation trials be required to collect adverse events from participants related to the evidence-based intervention even when risk is minimal? How should DSMBs operationalize monitoring for implementation trials, beyond trial accrual, dropouts, data quality, and missing data? What stopping rules should be implemented in this context? Most importantly, what is the evidence that DSMBs for implementation trials reduce risk to participants or improve data quality and trial integrity?

## Risk to Organizational Participants in Implementation Trials

Implementation trials are often conducted at the organization or practice level through cluster randomization and may pose potential risk to organizational participants [18–20]. These may be indirect and collateral participants in pragmatic clinical trials that are directly affected by the implementation of the interventions [21]. Smalley defines “indirect participants” as “…[I]ndividuals who are (1) not identified as direct participants and (2) whose rights and welfare may be affected by the intervention through their routine exposure to the environment in which the intervention is being deployed” and “collateral participants” as “[P]atient groups and other stakeholder communities who may be otherwise affected by the occurrence and findings of the pragmatic clinical trial” [21]. For example, the implementation of an intervention or practice could divert attention and resources, leading to potentially diminished access or quality in other areas [22].

An implementation trial could potentially adversely impact workflow and workforce, wherein clinicians and staff may be indirect participants during a study and collateral participants during broader dissemination of study results. In theory, these organizational-level harms could be monitored using routinely gathered administrative data or through data collected during the implementation processes, such as quality metrics not related to the study, staff turnover, and patient access. This is a specific example of the more general principle of the “unanticipated consequence of purposive social actions” [23]. Significant differences in any of these measures between those randomized to different treatment arms may require *a priori* development of early termination decision rules similar to those carried out at the patient level [24], with interim monitoring for these types of organizational- and staff-level adverse outcomes. However, actionable DSMB decisions are likely limited by statistical power for organizational-level events, unique contextual factors, few validated measures of organizational harm, and stepped wedge designs involving staggered rollout that hinder real-time direct comparisons based on actionable data. Moreover, groups and organizations are not considered research subjects under the Common Rule [4], possibly excluding them from oversight by DSMBs who are charged with protection of individual participants. Table 1 summarizes the challenges for DSMBs and future considerations.

**Table 1.** Potential focus of DSMB monitoring, challenges, and next steps

Focus of monitoring	Challenges	Next steps
Individual participants	Risk may be minimal without need for more intensive monitoring of individual participants. Implementation trials typically do not collect data on safety or adverse events related to the evidence-based practice. Overwhelming benefit or futility will be difficult to assess.	Clarification of the role (if any) of DSMBs for implementation trials where individual participant risk is minimal.
Data quality and trial integrity	Beyond periodic review of trial accrual data and missing data, it is not clear what DSMBs might monitor in this context. Implementation trials typically collect data on multiple implementation outcomes often based on mixed methods. It is not clear how DSMBs would develop stopping rules based on these complex assessments. Stepped wedge designs involve both within and between wedge comparisons, further complicating trial integrity monitoring.	Clarification of the role of DSMBs in terms of monitoring data quality and trial integrity for implementation trials. What if any metrics are feasible and appropriate for DSMBs to monitor?
Groups and organizations	Organizational harms exist and should be monitored and reported per CONSORT. However, the Common Rule only covers individual participants, not groups.	These harms should be considered in planning and assessed based on CONSORT guidelines for pragmatic and/or cluster trials [25,26], but fall outside the Common Rule and DSMBs. Research is needed on best practices for selection of relevant measures for assessing potential harms to groups.
Indirect participants [21]	These participants could include staff who are not directly consented or targeted by the implementation trial, but who are indirectly affected by changes in workflow or work burden. Families could be affected by organizational-level interventions. Future patients could be affected by strategies targeting clinicians or processes. Indirect participants are not covered.	These impacts should be considered in the design of implementation trials but are unlikely to be addressable by DSMBs. Further research is needed on best practices for selection of measures related to adverse effects on indirect participants.
Collateral participants [21]	Patients and organizational and other stakeholder are often involved in implementation trials through CBPR. Some of these participants may be individual participants and covered under the Common Rule, others are not covered.	Participants protected under the Common Rule (i.e., they qualify as individual participants) are protected in the same way as other individual participants. Guiding ethical principles for CBPR [27,28] could provide some protection to participants when these principles are followed. However, to date, there is typically no means for external monitoring of enactment of these principles.

CBPR, community-based (engaged) participatory research; DSMB, data and safety monitoring board.

## Reflections Going Forward

To spark discussion, we make the following suggestions. First, we suggest that NIH clearly distinguishes between implementation trials and phase III clinical trials of effectiveness to minimize any potential confusion among investigators regarding the scope of NIH policy on DSMBs.

Second, we suggest that NHLBI amend its current policy that universally requires DSMBs for multisite, implementation trials, and those funded through contracts and cooperative agreements. Given the dearth of data regarding the role of DSMBs for implementation trials, we propose that NHLBI defers to local institutional review boards to make individual determinations based on the justifications within proposals to ensure that DSMB appointment is commensurate with the risk, size, and complexity of the specific implementation trial. Since this determination is likely to be made after funding, we propose that NHBLI sets aside additional funding for DSMBs.

Third, we suggest that NIH promote the collection and monitoring of data addressing potential, unintended consequences for indirect and collateral participants, such as organizations and staff participating in implementation trials. Such data would inform the potential role for DSMBs in implementation trials, in addition to informing the science regarding not only the benefits, but also the potential harms of implementation strategies. Last, we encourage NIH to fund studies explicitly designed to assess whether DSMBs affect safety, data quality, or trial integrity in the context of implementation trials, including potential cost-effectiveness and cost–benefit analyses that could inform future DSMB policy and training.
